# First 2-year experience of nationwide newborn screening for severe forms of T and B cell immunodeficiency: 2.3 million newborns analyzed using TREC and KREC in Russia

**DOI:** 10.3389/fimmu.2026.1742811

**Published:** 2026-02-06

**Authors:** Andrey Marakhonov, Anna Mukhina, Irina Efimova, Natalia Balinova, Maria Ampleeva, Anastasia Bobreshova, Yulia Rodina, Dmitry Pershin, Viktoriia Zabnenkova, Oxana Ryzhkova, Zhanna Markova, Nadezhda Shilova, Ilya Zhanin, Kirill Savostyanov, Svetlana Matulevich, Fanil Bilalov, Alexander Koroteev, Andrey Donnikov, Dmitry Trofimov, Tatyana Bairova, Gulnara Seitova, Sergei Mordanov, Elena Nikolaeva, Zareta Esmurzieva, Elena Skorobogatova, Lyudmila Olkhova, Larisa Vakhonina, Daria Kostenko, Gleb Bronin, Sergey Zimin, Tatiana Bykova, Dmitry Balashov, Rena Zinchenko, Nikolai Grachev, Sergey Voronin, Anna Shcherbina, Sergey Kutsev

**Affiliations:** 1 Research Centre for Medical Genetics, Moscow, Russia; 2 Dmitry Rogachev National Medical Research Center of Pediatric Hematology, Oncology and Immunology, Moscow, Russia; 3 Federal State Autonomous Institution (FSAI) «National Medical Research Center for Children’s Health» of the Ministry of Health of the Russian Federation, Moscow, Russia; 4 S.V.Ochapovsky Regional Clinical Hospital №1, Krasnodar, Russia; 5 Republican Medical Genetic Centre, Ufa, Russia; 6 Diagnostic Centre (Medical Genetic), Saint-Petersburg, Russia; 7 National Medical Research Center for Obstetrics, Gynecology and Perinatology named after Academician V.I. Kulakov, Moscow, Russia; 8 Scientific Centre for Family Health and Human Reproduction Problems, Irkutsk, Russia; 9 Tomsk National Research Medical Center of the Russian Academy of Sciences, Tomsk, Russia; 10 Rostov State Medical University, Rostov-on-Don, Russia; 11 Clinical Diagnostic Centre «Mother and Child Healthcare», Yekaterinburg, Russia; 12 Morozovskaya Children’s City Clinical Hospital of the Moscow City Health Department, Moscow, Russia; 13 Russian Children’s Clinical Hospital of the N.I. Pirogov Russian National Research Medical University of the Ministry of Healthcare of the Russian Federation, Moscow, Russia; 14 Pediatric Oncology & Hematology Center, Regional Children’s Hospital, Yekaterinburg, Russia; 15 Raisa Gorbacheva Memorial Research Institute for Pediatric Oncology, Hematology and Transplantation, Pavlov First Saint Petersburg State Medical University, Saint-Petersburg, Russia

**Keywords:** agammaglobulinemia, inborn errors of immunity, KREC, newborn screening, primary immunodeficiency, severe combined immunodeficiency, TREC

## Abstract

**Introduction:**

Here, we present the results of a nationwide newborn screening (NBS) program in Russia, covering over 2.3 million newborns and employing TREC and KREC quantification to improve the identification of severe forms of T and/or B cell immunodeficiencies and enable early treatment initiation.

**Methods:**

A two-tier PCR testing strategy was used to define the screen-positive cohort, followed by confirmatory flow cytometry and genetic diagnostics, including fluorescent in situ hybridization (FISH) and whole-exome sequencing (WES).

**Results:**

A total of 191 patients were diagnosed with defined forms of primary immunodeficiencies (PID), encompassing several groups of inborn errors of immunity (IEI): severe combined immunodeficiency (SCID), agammaglobulinemia, combined immunodeficiency less severe than SCID, and syndromic forms of PID. The overall birth prevalence of severe forms of T and/or B cell immunodeficiencies was 1 in 12,298 live births (95%CI: 1:10,672–1:14,247), corresponding to 8.13 cases per 100,000 newborns (95%CI: 7.02–9.37). Although the positive predictive value of KREC-based screening was relatively low, its use enabled the detection of a substantial proportion of patients with syndromic forms of PID, including Nijmegen breakage syndrome and ataxia–telangiectasia, along with various forms of agammaglobulinemia. Interestingly, 16% of diagnosed newborns had a positive family history, often with previously undiagnosed affected siblings or parents. Additionally, a considerable number of newborns detected by NBS presented with syndromic disorders not currently classified as IEI, suggesting potential avenues for future expansion of the IEI list.

**Discussion:**

Importantly, early diagnosis through NBS allowed for the timely initiation of disease-specific treatments, including hematopoietic stem cell transplantation (HSCT), immunoglobulin replacement therapy, and targeted immunosuppressive or supportive care strategies. Early intervention may reduce the risk of severe infections, improve neurodevelopmental outcomes, and prevent irreversible organ damage or malignancies in predisposed syndromes. Overall, our study demonstrates the effectiveness of large-scale implementation of TREC/KREC-based NBS in identifying a broad spectrum of immunodeficiencies and highlights future directions for improving NBS algorithms, follow-up protocols, and individualized medical management for affected infants.

## 1 Introduction

As of the end of 2024, more than 40 countries worldwide have implemented newborn screening (NBS) for severe forms of T cell immunodeficiency/lymphopenia, based on the analysis of TREC (T cell receptor excision circles)—a byproduct of V(D)J-recombination of T-cell receptor genes that happened during T-cell differentiation within the thymus (1). These programs include national, regional, or pilot projects (2) and primarily aim at the identification of infants with severe combined immunodeficiency (SCID)—the most severe form of primary immunodeficiency (PID), or inborn errors of immunity (IEI). SCID is life-threatening unless immediate treatment, usually hemopoietic stem cell transplantation (HSCT), is provided in infancy (3).

IEI with low B cell numbers (agammaglobulinemia and others) (4) can also benefit from early diagnosis and implementation of immunoglobulin replacement therapy (IRT) and are potentially screenable using KREC (kappa-deleting recombination excision circles) analysis (5). Although there are discussions about the potential benefits of this analyte in screening panels, many countries have not adopted it due to perceived high referral rates and insufficient evidence of the cost-effectiveness and health benefits ratio (6). Thus, while T cell lymphopenia screening has seen significant global adoption, the implementation of screening for agammaglobulinemia remains limited to pilot projects (7, 8) and is under consideration in various regions.

After a successful pilot project conducted in 2022 (9), in 2023, Russia initiated a nationwide expanded newborn screening program (10) that, among other genetic conditions, included severe forms of T and B cell primary immunodeficiencies, utilizing TREC and KREC detection via PCR-based techniques.

Here we present the results of the first 2 years of the all-Russian NBS for PID using TREC and KREC.

## 2 Materials and methods

### 2.1 NBS program

Written parental informed consent was obtained for all newborns enrolled in the NBS program.

Dried blood spots were obtained on the 2^nd^ day of life in case of term newborns and on the 7^th^ day of life in case of preterm newborns according to the national standards. Preterm birth was defined according to the WHO criteria as delivery occurring before 37 completed weeks of gestation (11).

Age terminology during the perinatal period was used according to the American Academy of Pediatrics recommendations (12).

**Figure 1:**
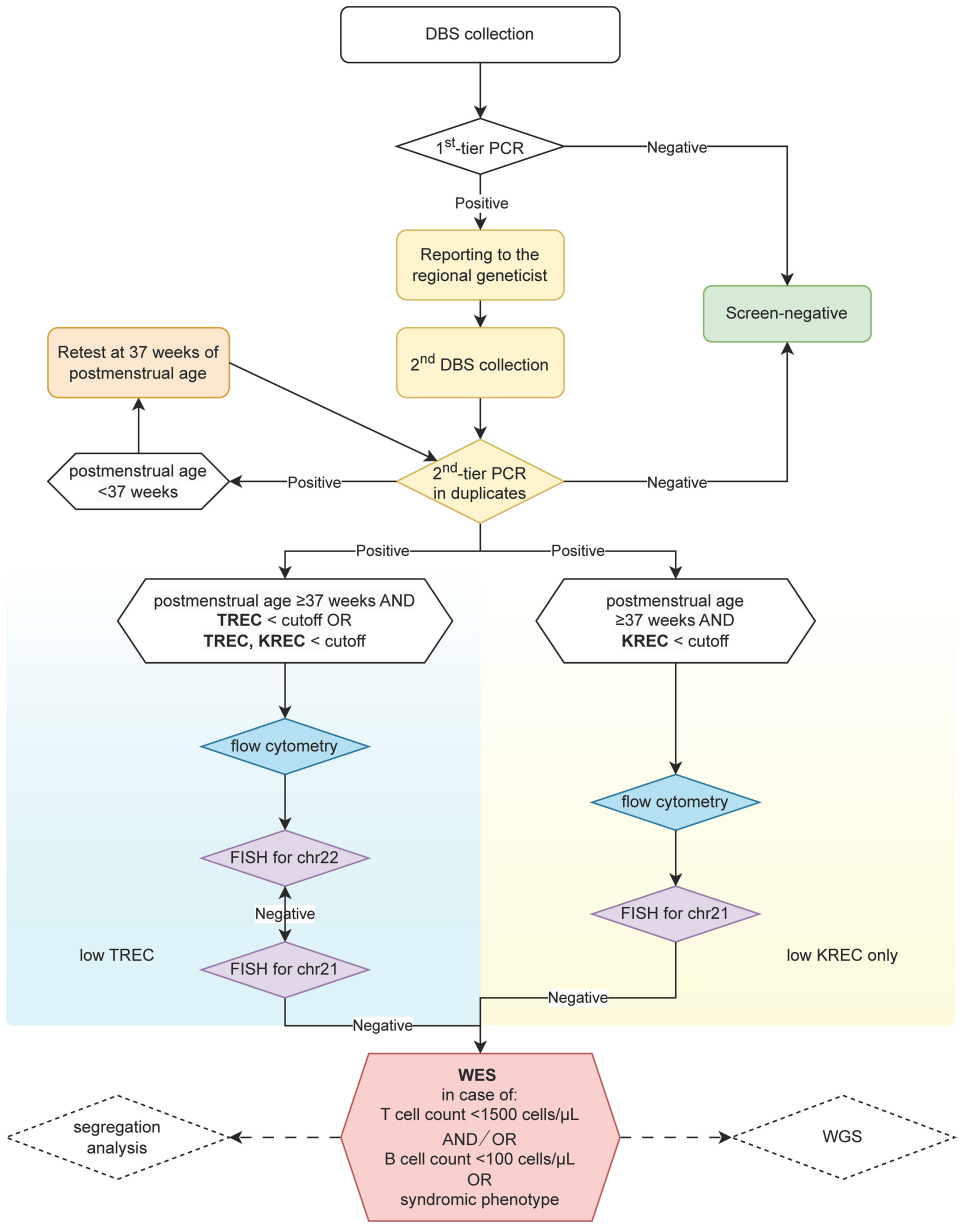
NBS workflow. FISH for chr21 was included in the workflow since 2024.

The workflow for the screening for PID in Russia is depicted in **Figure 1**.

First-tier PCR was performed at 10 centers for expanded newborn screening: one located in each of the big cities (Saint-Petersburg, Krasnodar, Ufa, Tomsk, Irkutsk, Rostov-on-Don, Yekaterinburg) and three located in Moscow (at the Morozovskaya Children’s City Clinical Hospital; at the National Medical Research Center for Obstetrics, Gynecology and Perinatology named after Academician V.I. Kulakov; and at the National Medical Research Center for Children’s Health of the Ministry of Health of the Russian Federation) (10). Two commercial multiplex qPCR-based test systems were used at the first-tier stage: TK-SMA kit (ABV-Test LLC, Moscow, Russia) and NeoScreen SMA/TREC/KREC REAL-TIME PCR Detection Kit (DNA-Technology TS, LLC, Moscow, Russia) according to the manufacturers’ recommendations. First-tier PCR cutoffs were set at 100 copies/10^5^ cells.

The second-tier PCR test was carried out in duplicates as described earlier (9). Briefly, the NBS tests were conducted utilizing the Eonis™ SCID-SMA kit (Wallac Oy, Turku, Finland) on the JANUS Extraction instrument (Perkin Elmer, Turku, Finland). Subsequently, real-time PCR analysis was performed using Applied Biosystems QuantStudio 5 Dx instruments (Thermo Fisher Scientific, Waltham, MA, USA), according to the manufacturer’s guidelines and recommendations. Second-tier PCR cutoff was determined according to the separate study published elsewhere (13).

Zero copies of TREC/KREC per 10^5^ cells were represented as 0.001 copies/10^5^ cells in figures and in statistical analysis.

Subsequent immunological confirmation of the PID diagnosis was performed according to the lyse-no-wash manufacturer’s protocol (Beckman Coulter, US) for multi-color flow cytometry method, using a Beckman Coulter CytoFLEX flow cytometer and a custom dry format DURA Innovations antibody panel (LUCID product line, Beckman Coulter, US) at the Dmitry Rogachev National Medical Research Center of Pediatric Hematology, Oncology and Immunology, (Moscow, Russia) as described earlier (9).

PID diagnosis was established according to the ESID (European Society for Immunodeficiencies) diagnostic criteria (14). SCID diagnosis was established according to the Primary Immune Deficiency Treatment Consortium (PIDTC) 2022 Definitions (15).

FISH analysis was performed on direct interphase nuclei preparations from whole blood collected in EDTA tubes. Deletion of the chromosome 22q11.2 region is determined by FISH, using the dual-color *TBX1* (22q11) (Spectrum Red) and *SHANK3* (22q13) (Spectrum Green) locus-specific probe (Leica Biosystems, Kreatech, US) for the DGS region according to the manufacturer’s recommendations. Trisomy 21 was detected using the dual-color *RCAN1* (Spectrum Red)/*RB1* (Spectrum Green) locus-specific probe (Leica Biosystems, Kreatech, US).

Whole-exome sequencing (WES) was performed on genomic DNA samples by targeted high-throughput sequencing (HTS) on the DNBSEQ-400 instrument in 2×151 bp paired-end mode. Further processing was performed as described elsewhere (16). Causative variants discovered by WES were validated by Sanger sequencing in the patient and parents, where appropriate. If no causative variants were detected, the whole-genome sequencing was performed when available.

As described earlier, the PID patient’s information was documented in the Russian PID registry maintained by the National Association of Experts in PID (NAEPID) (17).

### 2.2 Statistical analysis

The data collected during the study were analyzed using GraphPad Prism 8.0.1 (GraphPad Software, San Diego, California, USA). Throughout the analysis, data were presented as median with interquartile range (IQR), unless specified otherwise. Fisher’s 95% confidence interval (95%CI) for proportional data was computed using WinPepi v. 11.65 software (18).

The workflow was constructed with https://app.diagrams.net/. A Sankey diagram was constructed with https://sankeymatic.com/build/.

## 3 Results

### 3.1 NBS statistics

In total, 2,380,662 infants were born in Russia in the first 23 months since the implementation of NBS for PID. 2,348,872 samples were analyzed in the 1^st^-tier PCR stage in 10 centers responsible for NBS, resulting in 98.66% coverage (95%CI: 98.65–98.68%). 8,329 newborns were screen-positives at the 1^st^-tier PCR stage (0.35%; 95%CI: 0.35–0.36%).

Of them, 7,208 DBS samples were received for 2^nd^-tier PCR in RCMG (86.54%; 95%CI: 85.79–87.27%). The male-to-female ratio is 134.53:100.00, which is significantly higher than 106.78:100.00 in the general population (p-value = 4.50×10^-21^, χ²-test). Of them, 84.49% were DBS from term newborns (95%CI: 83.73–85.41%) while 13.96% were DBS from preterm newborns (95%CI: 13.16–14.78%). The proportion of preterm infants in the sample group was significantly higher than 5.94% (95%CI: 5.81–6.08%) in the general population (p-value = 1.05×10^-5^, χ²-test). The results of 2^nd^-tier PCR in full-term newborns were obtained at a median age of 19 days (IQR: 15–27).

**Figure 2:**
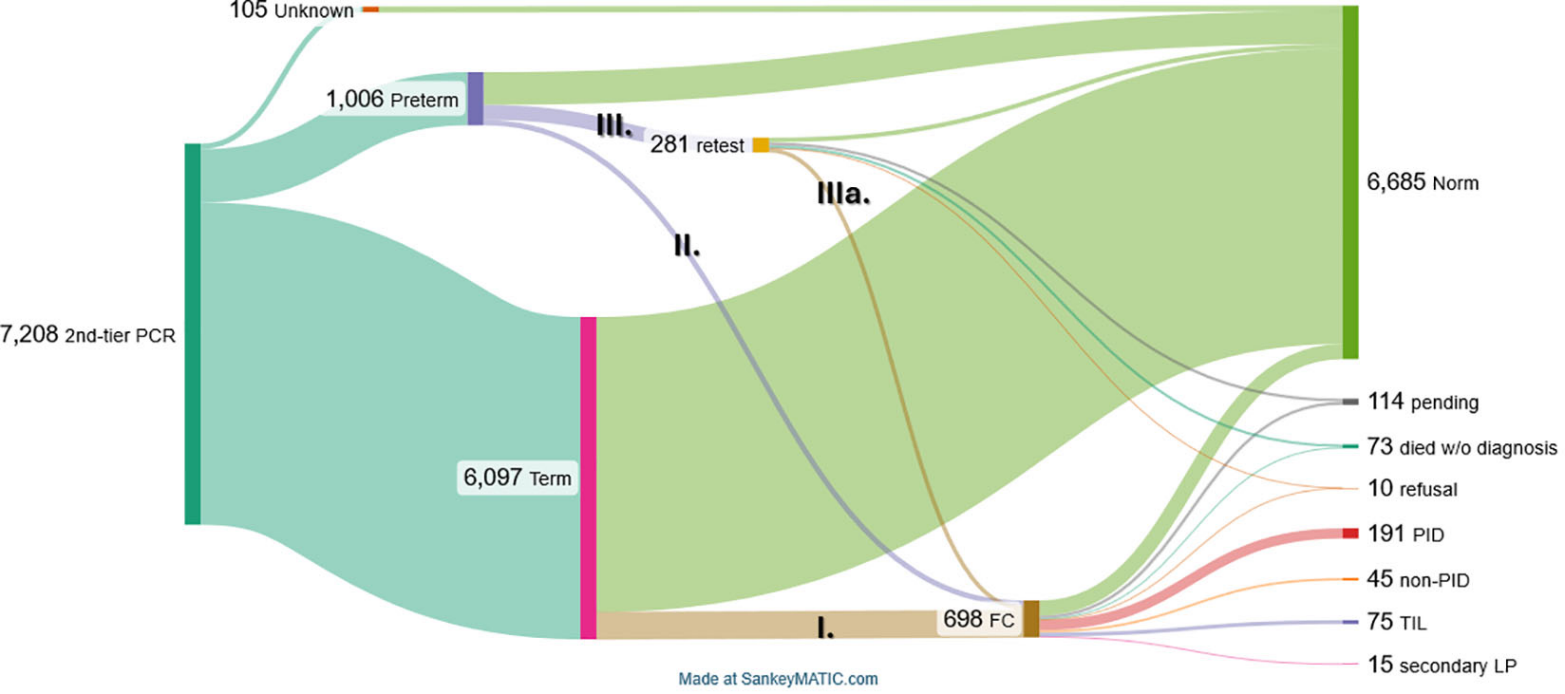
Sankey chart of sample processing at the reference center stage. Roman numbers (I–IIIa) represent stages depicted in detail in **Figure 3**.

At the 2^nd^-tier PCR stage, the vast majority of samples demonstrated TREC and/or KREC values above the cutoff (n=6314, 87.54%; 95%CI: 86.76–88.28%) while 898 samples were considered abnormal (12.46%; 11.70–13.24%) (**Figure 2**).

**Figure 3:**
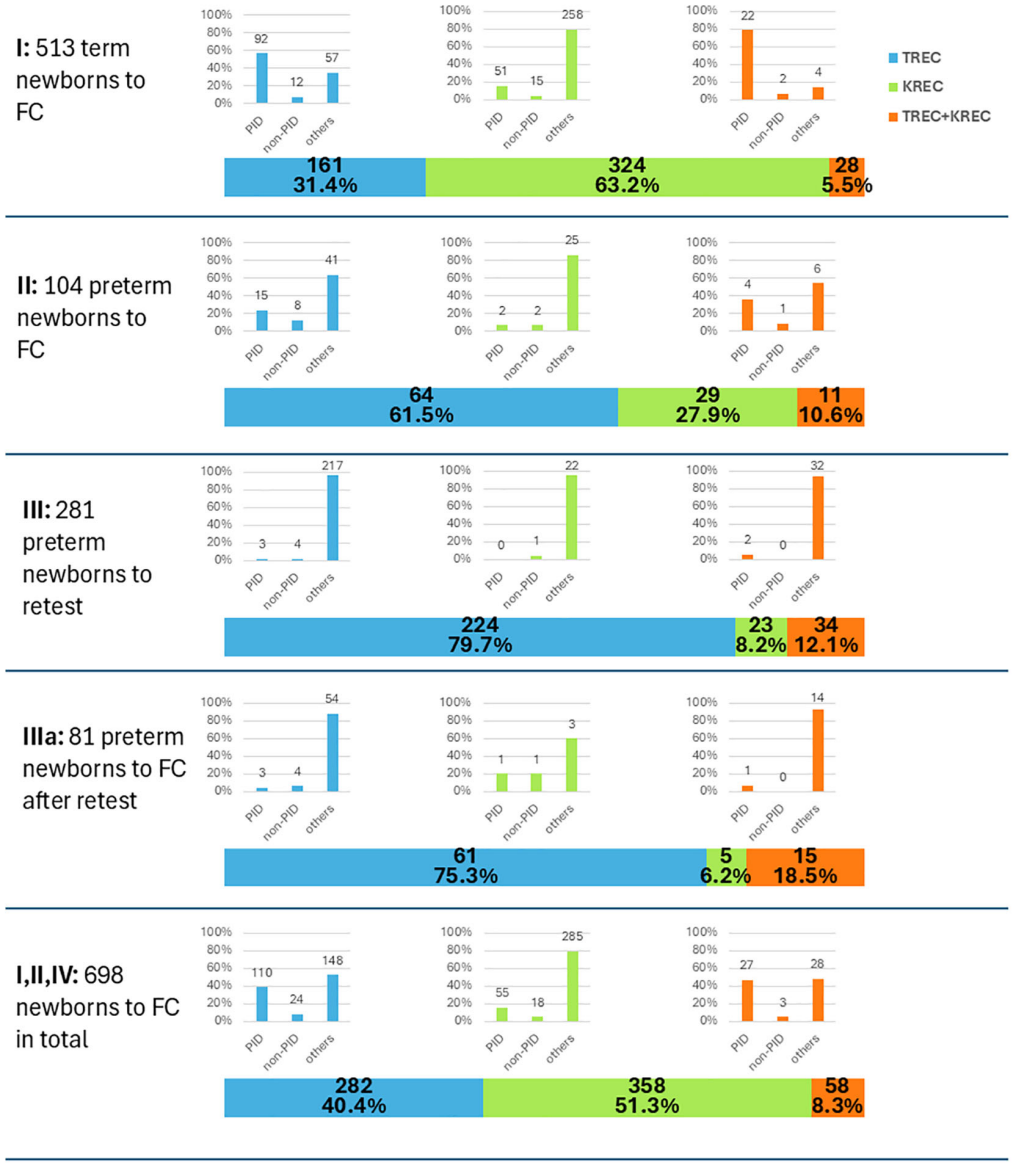
The distribution of abnormal results in either analyte according to the postmenstrual age and further steps. Roman numbers (I–IIIa) represent stages depicted in **Figure 2**. Distribution of newborns with abnormal TREC, KREC, or combined TREC+KREC results is shown in the horizontal bar plot, while the true-positive rate within each subgroup is depicted in the small inset vertical bar plots.

Newborns with abnormal screening results were classified into four groups (I, II, III, and IIIa) (**Figure 2**). We evaluated two key parameters in these groups, as illustrated in **Figure 3**: (i) distribution of newborns with abnormal TREC, KREC, or combined TREC+KREC results; and (ii) the true-positive rate within each subgroup. The true-positive rate was defined as the proportion of confirmed PID cases relative to the total number of newborns in each subgroup.

Group I consisted of term newborns who were immediately referred for immunophenotyping by flow cytometry. In this group, KREC deviations were more common than TREC or combined TREC/KREC abnormalities (**Figure 3**). However, the true-positive rate for KREC abnormalities was relatively low (15.7%) compared to TREC (57.1%) and combined TREC/KREC abnormalities (78.6%).

Group II included preterm newborns who showed abnormal results of the second-tier PCR test and had reached 37 weeks of postmenstrual age at the time. These infants were also referred for flow cytometry. As expected, TREC abnormalities were most frequent (61.5%), followed by KREC and TREC/KREC abnormalities (**Figure 3**). The true-positive rates were lower than in term newborns: 23.4% for TREC, 6.9% for KREC, and 36.4% for combined TREC/KREC abnormalities.

Group III consisted of preterm newborns who had abnormal second-tier PCR results but had not yet reached the postmenstrual age of 37 weeks. These infants were additionally tested at 37 weeks. Among the 281 preterm infants with abnormal initial results, 52 (18.51%; 95%CI: 14.14–23.55%) died before the follow-up test could be performed at 37 weeks of postmenstrual age. Two families declined further testing, and 57 infants had not yet reached 37 weeks of postmenstrual age at the time of analysis. Of those who underwent retesting, 89 (31.67%; 95%CI: 26.27–37.46%) exhibited normalization of TREC and/or KREC levels upon reaching 37 weeks: initially abnormal TREC results normalized upon retesting in 69.6% cases, as did most KREC abnormalities (in 88.0% cases); for the combined TREC/KREC group, 70.6% of cases were normal upon retesting. The remaining after retesting 81 preterm newborns (28.83%; 95%CI: 23.60–34.50%) had persistently low TREC and/or KREC levels, prompting referral for flow cytometry (FC) evaluation (Group IIIa, **Figure 2**). This Group IIIa had a relatively low true-positive rate: 4.9% for TREC, 20% for KREC, and 6.6% for combined TREC/KREC abnormalities (**Figure 3**).

Thus, 513 term newborns (group I), 104 preterm newborns after the 2^nd^-tier PCR stage (group II), and 81 preterm newborns after retesting at the 37^th^ week of postmenstrual age (group IIIa) were forwarded to the flow cytometry (**Figure 2**). The median time of flow cytometry analysis since the date of 2^nd^-tier PCR was 6 days (IQR: 4–8). Thus, the median age of FC analysis is 24 days for full-term newborns (IQR: 19–44.5), while for preterm infants it was 37 days (IQR: 24.5–72). Of 698 newborns, 311 demonstrated normal results of lymphocyte subpopulations analysis, and 286 (40.97%; 95%CI: 37.30–44.73%) were considered healthy and not followed up further. Despite normal FC results, samples of 25/311 were sent for genetic testing as syndromic features were observed in the infants. Of those, 14 were later found to have: Noonan syndrome due to heterozygous pathogenic variant in *PTPN11* gene in one, *USP9X*-linked syndrome in one, and various chromosomal syndromes in 12 (22q11.2DS in six; Wolf-Hirschhorn syndrome due to hemizygous deletion of chromosome 4p16.3 in one; 47,XYY syndrome in one; Down syndrome due to trisomy 21 in three, Edwards syndrome in one).

Of the 387 newborns who demonstrated abnormal results of immunophenotyping, 21 died prior to diagnosis verification (3.01%; 95%CI: 1.87–4.56%), and parents of 8 infants refused subsequent testing (1.15%; 95%CI: 0.50–2.25%). Fifteen newborns were diagnosed with secondary lymphopenia (LP; 2.15%; 95%CI: 1.21–3.52%): five due to exposure to immunosuppressive medications during pregnancy and ten due to chylothorax or hydrops fetalis. Seventy-five newborns (10.74%; 95%CI: 8.55–13.28%) were diagnosed with transient idiopathic lymphopenia (TIL) (**Figure 2**). These newborns underwent a whole spectrum of available immunologic and genetic tests that revealed no pathogenic genetic variants, and in whom subsequent restoration of lymphocyte subpopulation parameters was detected upon control FC at least one month after initial immunophenotyping.

### 3.2 PID revealed by screening

**Figure 4:**
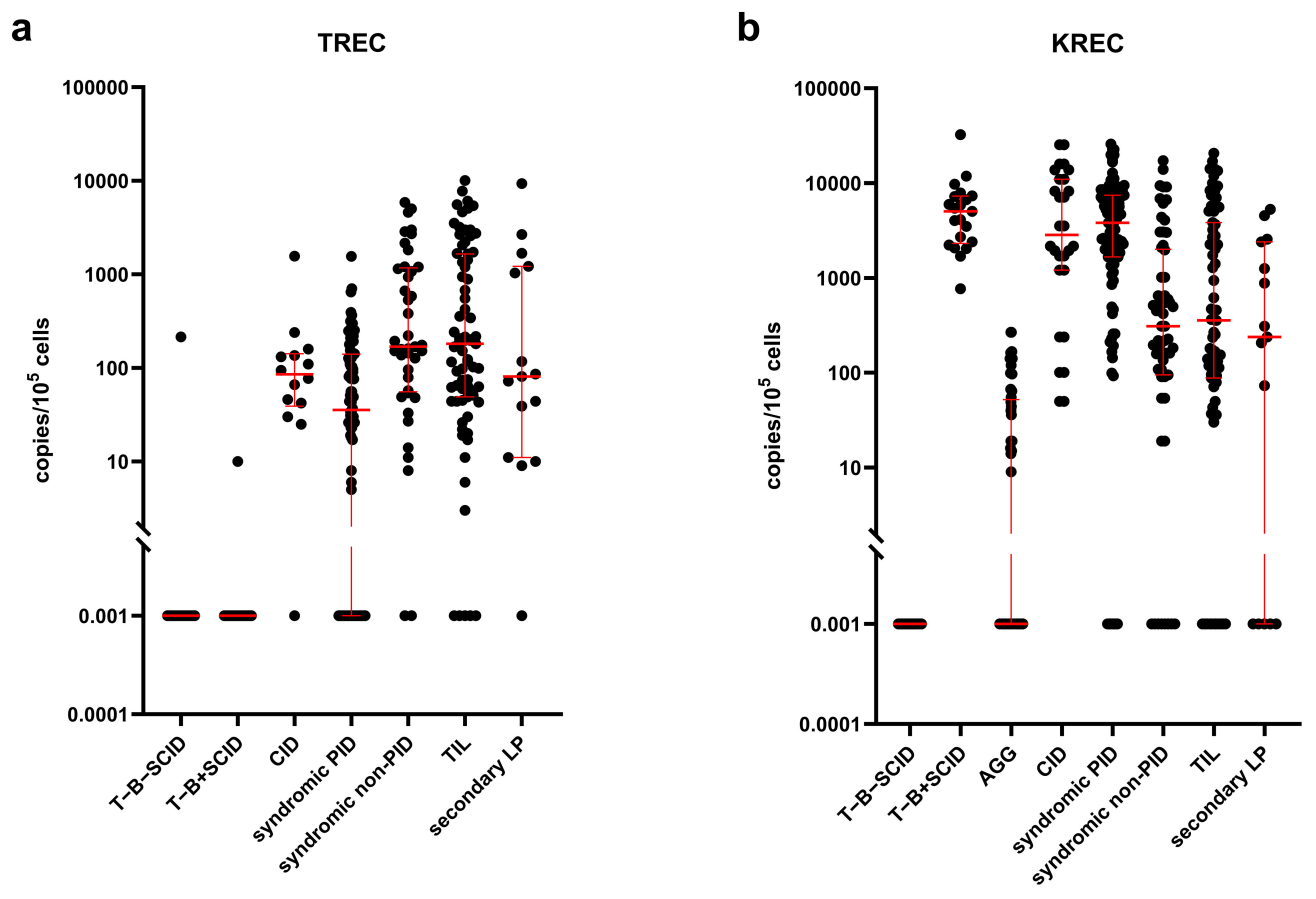
Distribution of TREC **(a)** and KREC **(b)** in patients detected through the NBS program. Red lines represent the median and interquartile range. Note the log₁₀ scale on the Y axis.

Finally, 191 newborns were diagnosed with various forms of primary immunodeficiency (27.36%; 95%CI: 24.09–30.83%). This resulted in severe forms of PID birth prevalence being 1:12,298 (95%CI: 1:10,672–1:14,247) or 8.13 cases per 100,000 live births (95%CI: 7.02–9.37 per 100,000 newborns). Their TREC and KREC values upon 2^nd^-tier PCR are shown in **Figure 4**.

**Figure 5:**
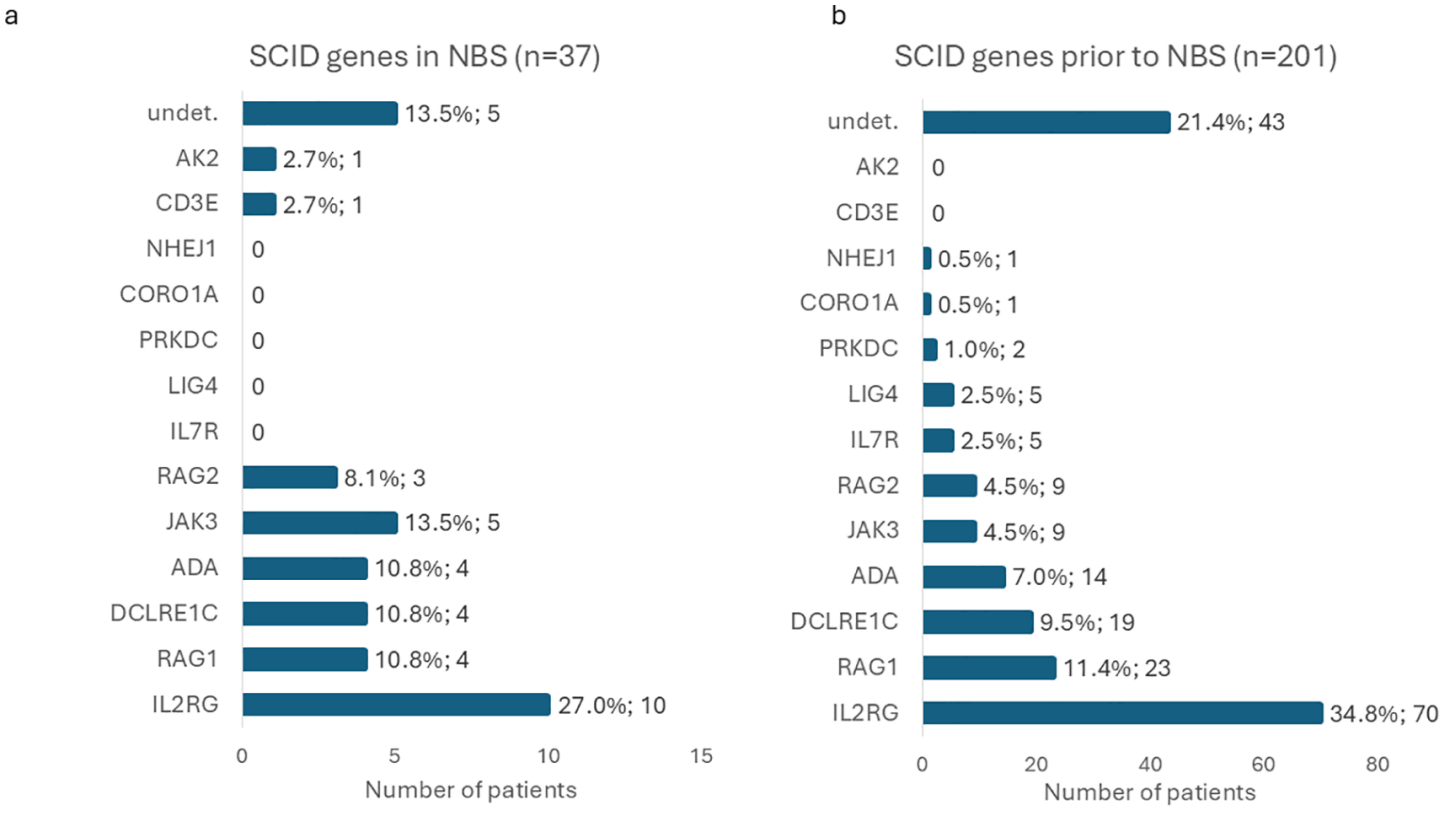
Distribution of causative genes identified in SCID patients detected through the NBS program **(a)** compared with those diagnosed prior to the implementation of NBS **(b)**. Horizontal bars represent the number of patients in each group.

Of these 37/191 newborns were diagnosed with SCID (**Supplementary Table S1**; **Figure 5**): 15 with T^-^B^-^SCID and 22 with T^-^B^+^SCID. Overall, the prevalence of SCID in Russia appeared to be 1:63,483 (95%CI: 1:46,057–1:90,163) or 1.58 cases per 100,000 live births (95%CI: 1.11–2.17 per 100,000 newborns). The most prevalent affected gene was *IL2RG* (27.03%; 95%CI: 13.79–44.12%), followed by *JAK3* (13.51%; 95%CI: 4.54–28.77%). Rarer defects were identified in *ADA* (n=4), *DCLRE1C* (n=4), *RAG1* (n=4), *RAG2* (n=3), *AK2* (n=1), and *CD3E* (n=1) genes. In SCID patients, 13 genetic variants were novel, and 27 have been previously described. Ten variants were found in a hemizygous state, 12 in a homozygous state, and 9 in a compound-heterozygous state. In 5 patients, no disease-causing variants were identified by WES; their samples are being tested by other methods at the time of writing (13.51%; 95%CI: 4.54–28.77%). Interestingly, one out of four patients with defects of the *ADA* gene was diagnosed due to absent KREC (0 copies/10^5^ cells), while the TREC level was above the cutoff (215 copies/10^5^ cells).

**Figure 6:**
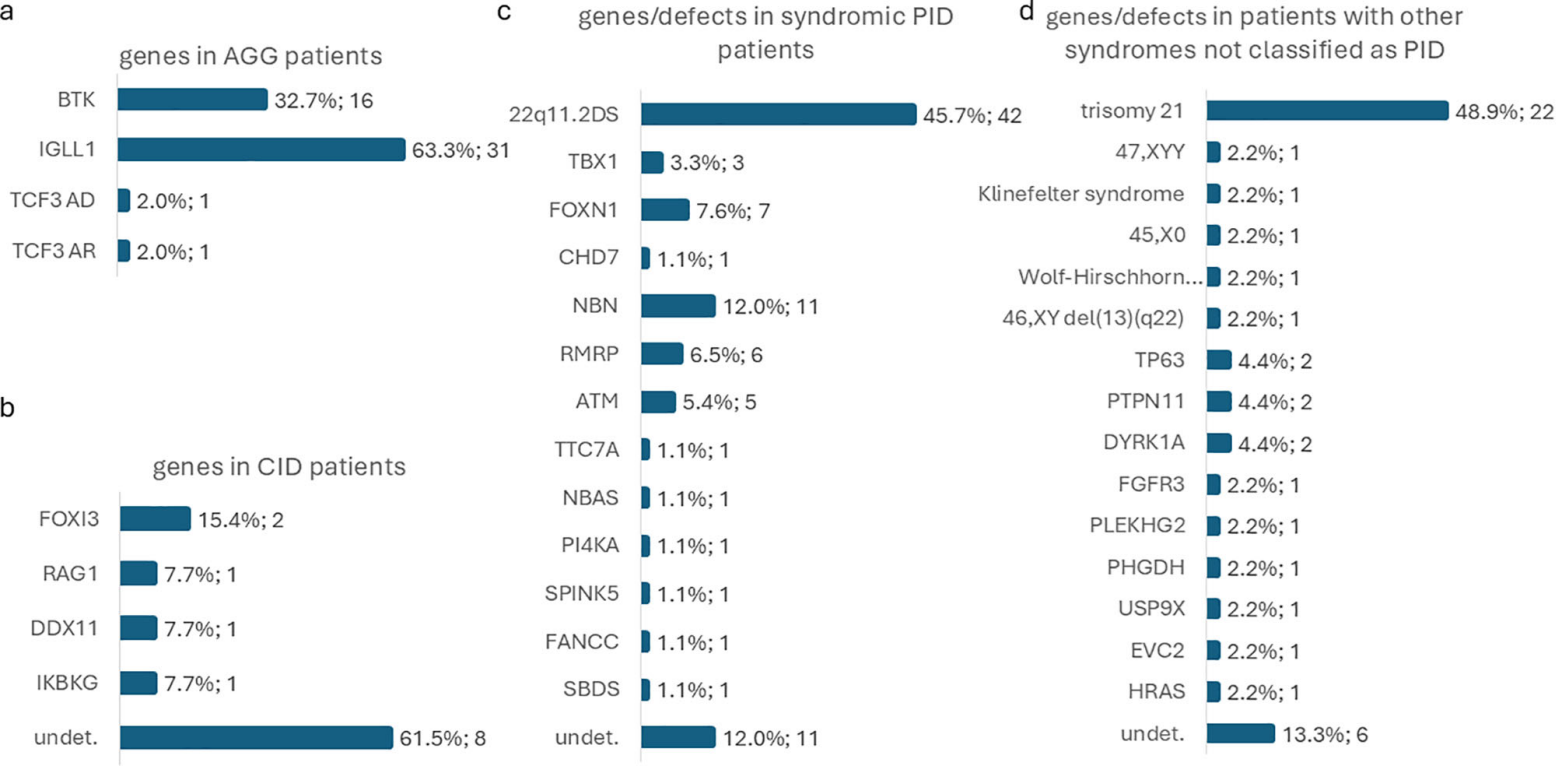
Distribution of causative genes identified in patients other than SCID within the NBS program: **(a)** B cell lymphopenia; **(b)** combined immunodeficiencies less severe than SCID; **(c)** syndromic forms of PID; **(d)** other syndromic conditions not currently classified as PID. Horizontal bars represent the number of patients in each group.

We have identified 49 patients with various defects attributed to agammaglobulinemia (AGG) (**Supplementary Table S2**; **Figure 6a**). In 16 patients, X-linked agammaglobulinemia (X-AGG) due to *BTK* defect (OMIM #300755) was confirmed, with 3 novel and 12 previously described variants identified. Overall, the prevalence of X-AGG in Russia appeared to be 1:146,804 (95%CI: 1:90,401–1:256,837) or 0.68 cases per 100,000 live births (95%CI: 0.39–1.11 per 100,000 newborns). One patient had a previously published *de novo* missense variant in the *TCF3* gene resulting in AD agammaglobulinemia type 8A (OMIM #616941). One more patient had a novel homozygous small deletion of 7 base pairs affecting the donor splicing site region of intron 7 in the *TCF3* gene, resulting in AR-AGG type 8B (OMIM #619824). Surprisingly, a large number of infants with low/absent KREC (n=31) were revealed to have biallelic variants in the *IGLL1* gene (OMIM #613500). In them, seven variants were found, three of which have been previously described in patients with confirmed AR agammaglobulinemia, and four were novel. *IGLL1* variants were found in a homozygous state in 18 patients, and in a compound-heterozygous state in 13 patients. Biallelic defects in *IGLL1* had a prevalence of 1:75,770 newborns (95%CI: 1:53,381–1:111,516) or 1.32 cases per 100,000 live births (95%CI: 0.90–1.87 per 100,000 newborns).

Combined immunodeficiency, less severe than SCID, constituted a significant group of PIDs found during NBS (n=13) (**Supplementary Table S3**; **Figure 6b**). Among them were patients with monoallelic *FOXI3* (n=2), hypomorphic *RAG1* (n=1), biallelic *DDX11* (n=1), and monoallelic *IKBKG* (n=1) variants; no causative defects were found after WES in 8/13.

A large number of PID patients had various forms of PID with syndromic features (n=92) (**Supplementary Table S4**; **Figure 6c**). The vast majority of them comprised 22q11.2 deletion syndrome (22q11.2DS) patients (n=42). Overall, the prevalence of 22q11.2DS identified in NBS is estimated to be 1:55,926 (95%CI: 1:41,374–1:77,597) or 1.79 cases per 100,000 live births (95%CI: 1.29–2.42). Three more patients were found to have novel single-nucleotide variants in the *TBX1* gene in a heterozygous state and had typical features of DiGeorge syndrome.

The second largest group of syndromic PIDs consisted of 11 patients with Nijmegen breakage syndrome, all having homozygous “Slavic” 5-nucleotide deletion in the *NBN* gene. The prevalence of Nijmegen breakage syndrome identified by NBS is estimated to be 1:213,534 (95%CI: 1:119,341–1:427,755) or 0.47 cases per 100,000 live births (95%CI: 0.23–0.84).

Seven newborns were revealed to have various novel heterozygous variants in the *FOXN1* gene. All of them demonstrated a phenotype of combined immunodeficiency with various syndromic features. In 4/7 cases, variants were inherited from an apparently healthy parent, in 1/7 it occurred *de novo*, and two more families were unavailable for segregation analysis.

Screening also revealed 6 patients with biallelic variants in the *RMRP* gene. They demonstrated lymphopenia of variable severity, ranging from SCID phenotype to mild lymphopenia. Ten distinct variants were identified; 2/10 were previously described, and the remaining were novel.

Five patients were diagnosed with ataxia–telangiectasia (AT) caused by biallelic variants in the *ATM* gene. Seven variants were identified; 2 of them were novel, and the remaining were previously described.

Seven more patients with syndromic forms of PID each had biallelic variants in *TTC7A*, *NBAS*, *PI4KA*, *SPINK5*, *FANCC*, *SBDS* genes, and one patient had a monoallelic variant in the *CHD7* gene. Eleven patients lacked potential causative variants after WES and remained genetically unverified at the time of publication.

### 3.3 Non-PID cases revealed by screening

Forty-six patients were found to have other genetic conditions not currently classified as PID (**Supplementary Table S5**; **Figure 6d**). The majority of cases were syndromes due to chromosomal aberrations and included 22 cases of trisomy 21, one case of 45,X, one case of 47,XYY syndrome, one case of Klinefelter syndrome, Edwards syndrome, Wolf-Hirschhorn syndrome, and deletion of the long arm of chromosome 13 syndrome. It is worth noting that in the first months of NBS, 10 cases of trisomy 21 were revealed during WES analysis since no FISH for chromosome 21 was originally performed. These cases of trisomy 21 revealed no other potential causative variants for lymphopenia. Later, we incorporated FISH for chromosome 21 in the first tier of genetic testing, and an additional 12 cases of trisomy 21 were confirmed. Other 12/46 cases were represented by monogenic syndromes with variants in genes *DYRK1A* (n=2), *PTPN11* (n=2), *TP63* (n=2), *HRAS*, *EVC2*, *USP9X*, *PHGDH*, *PLEKHG2*, and *FGFR3*. Genetic causes of 6 more cases are still under investigation.

### 3.4 Treatment of PIDs revealed by NBS

Thirty out of 37 SCID patients received HSCT, 23/30 (76,67%; 95%CI: 57.72–90.07%) are currently alive, at various stages post-HSCT. Two of the four ADA-SCID patients received enzyme replacement therapy prior to successful HSCT; details of their treatment will be reported in detail separately (Rodina et al, submitted). Of the seven patients who did not receive HSCT, one patient died before final diagnosis, the families of two patients refused HSCT, four were non-Russian citizens, and the infants and their families moved back to their countries of origin; all were lost to follow-up.

All 14 patients with combined immunodeficiency, less severe than SCID, demonstrated a need for immunoglobulin replacement therapy (IRT) and immunological follow-up. For two patients (*IKBKG* and *RAG1* deficiency), HSCT is being planned.

Six patients with syndromic forms of PID underwent curative therapies: one patient with complete DiGeorge syndrome underwent thymic transplant and unfortunately succumbed to severe CMV infection; two patients with Nijmegen breakage syndrome and 3 patients with *RMRP* defects underwent HSCT, all alive at different time points after the procedure. Initial recommendations for IRT were made in 48/91 cases of syndromic PIDs; the remaining cases are being followed by multidisciplinary teams.

All patients with XLA started IRT with IVIG and were subsequently switched to SCIG.

## 4 Discussion

We present the results of the first two years of an expanded NBS program for severe T and B cell immunodeficiencies, utilizing TREC and KREC quantification and encompassing over 2.3 million newborns. The screen-positive rate at the first-tier PCR stage was 0.35%, which is higher than previously reported data, such as the 0.12% rate observed in the Israeli program with a comparable number of screened newborns (p-value = 3.94×10^-6^, z-test for two proportions) (19). This is likely attributable to the inclusion of KREC measurement, as deviations in this parameter accounted for a substantial proportion of abnormal samples (**Figure 3**). The majority of these initially positive samples (87.54% for both TREC and KREC) were subsequently confirmed as normal at the second-tier PCR stage. This high rate of normalization may be attributed to the early timing of initial specimen collection—typically on the second day of life for term neonates—when TREC/KREC levels may still be physiologically lower (20). Additionally, technical errors during the first-tier PCR performed in regional laboratories cannot be excluded as a contributing factor. Improved accuracy was achieved with the collection of an additional DBS for second-tier PCR testing in the central laboratory.

Following multistep testing, 698 newborns were referred for FC, corresponding to a referral rate of 0.023‰. This rate is significantly lower than that reported in other national NBS programs, such as 0.15‰ in the Israeli program (p-value = 8.26×10^-14^, z-test for two proportions) (19). The confirmation rates varied for different groups of newborns, with those exhibiting isolated KREC abnormalities showing a significantly lower true-positive rate (15.4%) compared to those with TREC abnormalities (39.0%, p-value = 1.00×10^-8^, Fisher’s exact test) or combined TREC+KREC abnormalities (46.6%, p-value = 4.01×10^-7^, Fisher’s exact test). Higher false-positive rates associated with KREC analysis have been reported in several pilot studies (7, 9, 21). Nevertheless, KREC testing has proven valuable not only for identifying cases of X-linked agammaglobulinemia but also for detecting patients with Nijmegen breakage syndrome—particularly relevant for countries with substantial Slavic populations (22), as well as those with ataxia–telangiectasia (23). In addition, KREC-based newborn screening could facilitate the detection of other IEIs, such as hypomorphic (as in one of our *ADA* cases) or delayed-onset ADA deficiency (24) and Ikaros deficiency (25). Reduced KREC levels have also been observed in patients with trisomy 21, potentially guiding early supportive interventions in this population (26).

In total, 191 patients with PIDs were identified, yielding a birth prevalence of 1 in 12,298 live births (95%CI: 1:10,672–1:14,247), or 8.13 cases per 100,000 newborns (95%CI: 7.02–9.37). Despite near-complete coverage of the target population (98.66%), approximately 31,750 newborns were not screened and could account for an estimated 2–3 presumably missed cases of severe T and B cell immunodeficiencies. The observed birth prevalence of severe T and/or B cell immunodeficiencies is consistent with the results of our earlier pilot study (p-value = 0.5855, z-test for two proportions) (9). These figures are significantly higher than pre-NBS estimates of PID incidence in Russia, which stood at 5.7 cases per 100,000 live births (p-value = 0.0015, z-test for two proportions) (17)—a noteworthy observation, considering that these earlier estimates included all forms of IEIs, whereas NBS detects only a small subset. Forms of PID detected during the two years of NBS represent not more than 15% of all forms in the Russian registry, which potentially translates to all PID prevalence of 1 in 1,835.

SCID represents the primary target of NBS screening. In the present study, the estimated birth prevalence of SCID was 1:63,483 (95%CI: 1:46,057–1:90,163). Although this estimate is lower than that obtained in our prior pilot study (1:28,986 newborns; 95%CI: 1:14,084–71,428) (probably due to the relatively small cohort in the pilot study), the difference was not statistically significant (p-value = 0.0511, z-test for two proportions). The current estimate aligns more closely with global SCID prevalence figures (27, 28). Importantly, the observed SCID birth prevalence remains significantly higher than pre-NBS estimates reported in Russia, which indicated an incidence of 1:247,000 of live births (p-value = 1.26×10^-12^, z-test for two proportions) (17). We also compared the distribution of genes affected in SCID patients diagnosed before and after the implementation of NBS (**Figure 5**). Certain causative genes with very low frequency of defects were identified only in one cohort, but not the other: *AK2* and *CD3E* defects were identified only in the NBS cohort, while single cases of *NHEJ1*, *CORO1A*, *PRKDC*, *LIG4*, and *IL7R* were observed to date only in patients diagnosed prior to NBS implementation. Despite these differences, the overall gene distribution did not differ significantly between the two groups (p-value = 0.1370, likelihood-ratio χ²-test), though the NBS cohort had a tendency for a higher proportion of *JAK3* defects and a lower proportion of X-SCID. Statistically significant difference is also not observed when only confirmed cases were taken into account (p-value = 0.1390, likelihood-ratio χ²-test).

During the two years of the NBS program, no SCID patients were missed due to having a TREC/KREC above the cutoff and being diagnosed on the basis of clinical presentation. In one case, however, the diagnostic process was delayed as a result of organizational mistakes.

Post-HSCT SCID survival rate increased significantly form 0.56 (95%CI: 0.46–0.67) in the historical Russian cohort (29) to 0.7667 (95%CI: 0.5772–0.9007) (p-value = 0.0350), and not statistically significant than those of SCID patients identified in screening programs summarized by the Primary Immunodeficiency Treatment Consortium (PIDTC) (0.938, 95%CI: 0.879–0.968) (p-value = 0.0830) (30). Due to at least a fourfold increase in SCID incidence, the need for additional HSCT units in tertiary centers in Russia is obvious.

A substantial proportion of identified patients were diagnosed with agammaglobulinemia, made possible through the inclusion of KREC analysis in the NBS program. The overall incidence of X-linked agammaglobulinemia (XLA) was 1 in 146,804 live births (95%CI: 1:90,401–1:256,837), which is consistent with previously reported estimates (31). Among other findings, two hypomorphic *BTK* variants were identified, both associated with detectable, non-zero KREC levels. Unexpectedly, a larger number of patients with low KREC levels and confirmed B-cell lymphopenia were found to carry biallelic variants in the *IGLL1* gene. The description of these cases is beyond the scope of the present article and will be discussed in detail in a separate publication.

An important subgroup of patients included those with CID less severe than SCID. The rate of genetic confirmation in this group was 38.46%, significantly lower than that observed in the SCID group (72.97%; p-value = 0.0017). Nevertheless, several notable findings were observed. A case of hypomorphic *RAG1* deficiency exemplified a classic “leaky” SCID phenotype, involving a *de novo* missense *RAG1* variant (NM_000448.2:c.2299C>T, p.Pro767Ser) *in trans* with a maternally inherited previously described missense variant (NM_000448.2:c.1229G>A, p.Arg410Gln). Two heterozygous novel variants in the recently described *FOXI3* gene (32) were identified in patients with CID and mild craniofacial dysmorphisms consistent with recent observations (33). Previously reported *de novo* donor splice-site variant in exon 5 of *IKBKG* (NM_001099857.3:c.671+2T>G) (34) was found in a female patient in a state of somatic mosaicism presenting with CID, autoinflammatory features, and signs of incontinentia pigmenti. Another interesting case is a male patient harboring two variants in the *DDX11* gene who was unfortunately lost to follow-up. This group of patients requires close clinical monitoring and may benefit from targeted disease-specific management strategies beyond standard immunoglobulin replacement therapy (35).

A substantial proportion of PID cases were associated with syndromic forms, comprising 47.64% of patients (95%CI: 40.38–54.98%). Genetic confirmation rate in this group is relatively high (87.9%). Among these, 22q11.2 deletion syndrome (22q11.2DS) was the most prevalent condition. Notably, in 2 out of 42 cases (4.76%), the deletion was inherited from an apparently unaffected parent without a prior diagnosis. Immunological involvement among patients with 22q11.2DS identified through NBS varied significantly—from SCID-like T-cell lymphopenia to near-normal T cell counts. Such phenotypic variability is well recognized in 22q11.2DS (36). According to the literature, NBS can detect approximately 5–20% of all 22q11.2DS cases (37, 38), although this proportion depends strongly on the TREC cutoff values employed in the screening protocol (13). In addition, a monogenic form of TBX1 haploinsufficiency was identified; in 2 out of 3 cases, it was inherited from a clinically unaffected parent.

Not surprisingly, with the majority of the population of Russia being of Slavic origin, Nijmegen breakage syndrome caused by homozygous “Slavic” founder variant in *NBN* was the second most common diagnosis among patients with syndromic forms of PID. Although the observed birth prevalence of Nijmegen breakage syndrome in our cohort exceeded pre-NBS estimates, the difference was not statistically significant (p-value = 0.1584) (17). A positive family history was noted for one family, in which the older sister received the diagnosis only when her infant brother underwent NBS. It is plausible that the true incidence of Nijmegen breakage syndrome is underestimated because not all affected individuals may be detected by TREC-based screening alone (13). Nonetheless, the addition of KREC analysis proved valuable: 6 out of 11 patients diagnosed with Nijmegen breakage syndrome through the NBS program exhibited decreased KREC levels. Early detection of Nijmegen breakage syndrome through newborn screening could play a critical role in clinical management, particularly by facilitating timely HSCT, thereby reducing long-term cancer risk (39, 40). Moreover, low KREC numbers predict disease severity, as has been demonstrated previously (40). Hence, detection of low KREC in these infants may influence the decision to perform HSCT promptly, in the first year of life.

FOXN1 haploinsufficiency was identified in seven patients, all of whom exhibited varying degrees of T cell impairment, consistent with previous reports (41). Notably, in four of the five cases with available segregation data, the variants were inherited from apparently unaffected parents, supporting the observation that T cell function may improve with age in *FOXN1*-related immunodeficiency.

Cartilage–hair hypoplasia (CHH), caused by biallelic pathogenic variants in the *RMRP* gene, was diagnosed in six patients. CHH is known for its broad clinical and immunological variability (42), and our cohort similarly exhibited a spectrum of immunodeficiency, ranging from a SCID-like phenotype to mild lymphopenia. Importantly, NBS enabled a post-mortem molecular diagnosis to be made in a girl with a CID phenotype who died from diffuse large B-cell lymphoma at the age of three. She was the older sister of a female infant identified through the screening program, also with a SCID phenotype, who was successfully transplanted at the age of 6 months. As with other studies (43), all CHH patients with absent TRECs and a SCID/leaky SCID phenotype underwent transplantation due to urgent indications, except for one patient whose parents refused treatment. Other CHH patients with low TRECs and a CID phenotype receive immunoglobulin replacement therapy, antimicrobial prophylaxis, and are being monitored. It has been previously suggested (44) that HSCT decision in CHH patients with low TREC levels should be based on the dynamics of naïve T lymphocyte counts. However, given the high risk of malignancies and infectious complications (45), the timeline for HSCT in this cohort requires further investigation.

Ataxia–telangiectasia (AT) is a disorder with substantial variability in clinical presentation. Within the NBS program, five patients with AT were identified; notably, two were detected solely due to low KREC levels with TREC values substantially higher than 500 copies/10^5^ cells (**Figure 4**). Early diagnosis of AT through NBS may facilitate improved immunological and oncological outcomes (46). Although there is no curative treatment for this disease, an early diagnosis makes family counseling and prenatal diagnosis in subsequent pregnancies possible and necessary. We previously reported the median diagnostic delay in AT to be three years (range 0–14) (17), which led to some families having more than one affected child. Currently, 25 families (12.9%) with at least two affected individuals are listed in the Russian PID registry (unpublished, 2025).

Interestingly, other, unexpected forms of PID that also benefit from early diagnosis were detected via NBS. For instance, an infant with an isolated decrease of KREC was diagnosed with Schwachman–Diamond syndrome (**Figure 4**).

Positive family history emerged as a significant feature in our cohort. Among autosomal recessive forms of PID, it was observed in 4/39 cases (10.26%; 95%CI: 2.87–24.22%). Family history is recognized as one of the ten warning signs of primary immunodeficiencies (47). In populations with high rates of consanguineous marriages, a positive family history may be observed in up to 66% of cases (48). The proportion identified in our cohort aligns with values reported for other autosomal recessive disorders in population-based genetic epidemiological studies of the Russian population (49). Notably, X-linked forms of PID revealed by NBS exhibited a much higher rate of positive family history, accounting for 3/16 X-AGG cases and 3/9 X-SCID cases, totaling 24.00% (95%CI: 9.36–45.13%).

NBS enabled the identification of a substantial proportion of patients with syndromic conditions that are not currently classified as inborn errors of immunity (IEI). In some cases, the observed lymphopenia may be attributable to general immaturity or preterm birth; however, in others, it may represent a primary immunological defect. The latter is particularly likely when recurrent cases with pathogenic defects exhibit similar immunological profiles—as observed in two unrelated patients with *TP63* variants (50) or deletion of the long arm of chromosome 13 syndrome (51). We anticipate that such findings will contribute to the future expansion of the IEI classification as the number of similar cases continues to grow.

## 5 Conclusion

In conclusion, the nationwide implementation of NBS using TREC and KREC has led to significant improvements in the early identification of patients with severe forms of T and/or B cell immunodeficiencies, enabling the timely initiation of life-saving treatments. Moreover, the program has demonstrated the potential to refine and optimize NBS algorithms based on insights gained during the first two years of implementation. Importantly, NBS has also contributed to the identification of previously undiagnosed affected relatives within families, emphasizing its broader diagnostic impact. In addition, screening has expanded our understanding of immune system involvement in genetic disorders not currently classified as IEI, necessitating not only further studies of the disease, but also inclusion of an immunologist in a multidisciplinary team that follows such patients.

## Data Availability

The data that support the findings of this study are available on request from the corresponding author. The data are not publicly available due to privacy or ethical restrictions.

## Supplementary material

Supplementary Table 1 SCID patients diagnosed via NBS.

Supplementary Table 2 Agammaglobulinemia patients diagnosed via NBS.

Supplementary Table 3 Combined immunodeficiency patients diagnosed via NBS.

Supplementary Table 4 Syndromic PID patients diagnosed via NBS.

Supplementary Table 5 Syndromic patients not currently classified as PID revealed in NBS.
