# Supplementary material for: First 2-year experience of nationwide newborn screening for severe forms of T and B cell immunodeficiency: 2.3 million newborns analyzed using TREC and KREC in Russia
Source: Front Immunol. 2026 Feb 6;17:1742811. doi: 10.3389/fimmu.2026.1742811 (PMC12920452; doi:10.3389/fimmu.2026.1742811)
Supplement: Supplementary file 2 [file DataSheet1.docx]

Supplementary Material

# Russian National Newborn Screening team

**Belgorod Region**

- **Svetlana Vladimirovna Vinoglyadova**, St. Joasaph Belgorod Regional Clinical Hospital
- **Elena Alexandrovna Guryleva**, St. Joasaph Belgorod Regional Clinical Hospital
- **Irina Vladimirovna Smoleva**, Regional Pediatric Clinical Hospital, Belgorod

**Bryansk Region**

- **Elena Vasilievna Gerasyutina**, State Autonomous Healthcare Institution “Bryansk Clinical and Diagnostic Center
- **Tatyana Petrovna Alexandrova, Regional Pediatric Clinical Hospital, Bryansk**

**Vladimir Region**

- **Natalya Anatolyevna Irinina**, Vladimir Regional State Healthcare Institution “Regional Clinical Hospital”
- **Evgeniya Vladimirovna Ignatieva**, Vladimir Regional Pediatric Clinical Hospital

**Voronezh Region**

- **Tatyana Valeryevna Fedotova**, State Healthcare Institution “Voronezh Regional Clinical Hospital No. 1”
- **Natalya Borisovna Yudina**, Voronezh Regional Pediatric Hospital №1

**Ivanovo Region**

- **Tatyana Pavlovna Zhukova**, V.N. Gorodkov Ivanovo Research Institute of Maternity and Childhood, Ministry of Health of the Russian Federation

**Kaluga Region**

- **Inessa Alexandrovna Kuzmicheva**, Kaluga Regional Clinical Hospital, Perinatal Center
- **Elvira Nikolaevna Tyulyakova**, Kaluga Regional Clinical Pediatric Hospital

**Kostroma Region**

- **Ekaterina Mikhailovna Korzhina**, Maternity Hospital of Kostroma, Center for Family Health and Reproduction Protection

**Kursk Region**

- **Ekaterina Konstantinovna Vyalykh**, Centralized Medical Genetic Counseling Office, Kursk Regional Multidisciplinary Clinical Hospital, Kursk

**Lipetsk Region**

- **Inna Ivanovna Shcherbina**, Lipetsk Regional Perinatal Center

**Moscow Region**

- **Yulia Yuryevna Kotalevskaya**, Medical-Genetic Center, MONIKI
- **Ekaterina Sergeevna Nikonova**, Scientific Research Clinical Institute for Childhood, Department of Allergology and Immunology for Children

**Moscow City**

- **Yulia Vladimirovna Reshetova**, Morozovskaya Children's City Clinical Hospital of the Moscow City Health Department
- **Natalya Valentinovna Zinovyeva**, Speransky Children’s City Clinical Hospital № 9
- **Sergei Borisovich Zimin**, Morozovskaya Children’s City Clinical Hospital of the Moscow Healthcare Department
- **Ekaterina Igorevna Kovtun**, Morozovskaya Children's City Clinical Hospital of the Moscow City Health Department

**Oryol Region**

- **Svetlana Nikolaevna Stavtseva**, Z.I. Kruglaya National Clinical Medical Center, Oryol
- **Ivan Vladimirovich Fisyun**, Budgetary Healthcare Institution of the Oryol Region ‘Z.I. Kruglaya Scientific and Clinical Multidisciplinary Center for Maternal and Child Health Care’

**Ryazan Region**

- **Grigory Iosifovich Yakubovsky**, Ryazan Regional Clinical Perinatal Center
- **Vyacheslav Vyacheslavovich Lebedev**, Medical Clinics of Ryazan State Medical University named after Academician I.P. Pavlov

**Smolensk Region**

- **Natalya Viktorovna Lukina**, Clinical Hospital No. 1, Perinatal Center, Medical Genetic Counseling Office
- **Tatyana Mikhailovna Romankova**, Clinical Hospital No. 1, Perinatal Center, Medical Genetic Counseling Office
- **Elena Vladimirovna Volkova**, Smolensk Regional Children’s Clinical Hospital of the Smolensk Region Health Department

**Tambov Region**

- **Galina Alexandrovna Erbis**, Tambov Regional Children’s Clinical Hospital
- **Nadezhda Valeryevna Martynova**, Tambov Regional Children’s Clinical Hospital

**Tver Region**

- **Elena Mikhailovna Kochegurova**, Tver State Medical University, Ministry of Health of the Russian Federation

**Tula Region**

- **Tatyana Vladimirovna Yudintseva**, Tula Regional Perinatal Center

**Yaroslavl Region**

- **Vladislav Borisovich Velednitsky**, Yaroslavl Regional Perinatal Center
- **Olga Mikhailovna Laba**, Yaroslavl Regional Pediatric Hospital

**Arkhangelsk Region**

- **Maria Mikhailovna Rudaleva**, P.G. Vyzhletsov Arkhangelsk Regional Children’s Clinical Hospital
- **Alla Valeryevna Gorenkova**, Northern State Medical University” of the Ministry of Healthcare of the Russian Federation

**Vologda Region**

- **Nadezhda Georgievna Filatova**, Vologda Regional Clinical Hospital

**Kaliningrad Region**

- **Marina Anatolyevna Novik**, Regional Perinatal Center of the Kaliningrad Region
- **Aishat Ziyabutdinovna Markhaychuk**, Kaliningrad Regional Pediatric Clinical Hospital

**Republic of Karelia**

- **Ekaterina Viktorovna Tukhkanen**, V.A. Baranov Republican Hospital, Petrozavodsk
- **Maria Igorevna Borisova**, V.A. Baranov Republican Hospital, Petrozavodsk

**Republic of Komi**

- **Vera Nikolaevna Belyaeva**, Komi Perinatal Center

**Leningrad Region**

- **Anna Artemyevna Tsvetkova-Belousova**, Leningrad Regional Clinical Hospital
- **Elena Konstantinovna Kudryashova**, Leningrad Regional Clinical Hospital

**Murmansk Region**

- **Maria Nikolaevna Chebukhanova**, Murmansk Regional Clinical Hospital
- **Olga Vasilievna Sakhar**, Murmansk Regional Clinical Hospital
- **Ekaterina Igorevna Sulima**, Ministry of Health of the Murmansk Region

**Novgorod Region**

- **Lyudmila Vladimirovna Secheneva**, Novgorod Regional Children’s Clinical Hospital,

**Pskov Region**

- **Sergey Vladimirovich Solovyev**, Pskov City Hospital

**Saint Petersburg**

- **Anastasia Yuryevna Lobenskaya,** Saint Petersburg Medical-Genetic Diagnostic Center
- **Elena Andreevna Serebryakova**, Saint Petersburg Medical-Genetic Diagnostic Center
- **Olga Valeryevna Trusova**, Saint-Petersburg State Pediatric Medical University, Department of Hospital Therapy with a course in Allergology and Immunology named after M.V. Chernorutsky (Saint-Petersburg)
- **Olga Gennagievna Chernykh**, Saint-Petersburg, Consultative and Diagnostic Center for Children
- **Marina Nikolaevna Guseva**, Saint-Petersburg Pasteur Institute (Saint-Petersburg)
- **Aleksandra Leonidovna Laberko**, Saint-Petersburg, Raisa Gorbacheva Memorial Research Institute of Children Oncology, Hematology and Transplantation

**Nenets Autonomous Okrug**

- **Alexander Vasilyevich Kovyazin**, Nenets Autonomous Okrug

**Republic of Adygea**

- **Zarema Nalbievna Sakhtaryek**, Maykop City Clinical Hospital

**Astrakhan Region**

- **Irina Vladimirovna Soprunova**, Astrakhan Regional Center for Family Health and Reproduction
- **Irina Alexandrovna Sinelnikova**, Astrakhan Regional Center for Family Health and Reproduction
- **Elena Vladimirovna Krasilova**, Astrakhan Regional Pediatric Clinical Hospital

**Volgograd Region**

- **Ekaterina Evgenievna Shipovskova**, Volgograd Regional Clinical Hospital No. 1
- Tatyana Viktorovna Samofalova, Pediatric Polyclinic №2, Volgograd
- Kristina Borisovna Soldatova, Volgograd Regional Clinical Oncology Center

**Republic of Kalmykia**

- **Vera Erdneevna Ushanova**, O.A. Shungaeva Perinatal Center of the Republic of Kalmykia

**Krasnodar Krai**

- **Lyudmila Vasilyevna Zinchenko,** Ochapovsky Regional Clinical Hospital No. 1, Krasnodar
- **Saida Yusufovna Pseush,** Ochapovsky Regional Clinical Hospital No. 1, Krasnodar
- **Svetlana Anatolyevna Kortkhmazova**, Ochapovsky Regional Clinical Hospital No. 1, Krasnodar
- **Polina Dmitrievna Lashevich**, Ochapovsky Regional Clinical Hospital No. 1, Krasnodar
- **Eleonora Stanislavovna Ilyina**, Children’s Regional Clinical Hospital, Krasnodar

**Rostov Region**

- **Alla Mikhailovna Loboda, Rostov State Medical University**
- **Olga Anatolievna Areshina, Rostov State Medical University**
- **Maria Aleksandrovna Amelina**, Regional Children’s Clinical Hospital of Rostov Region
- **Olga Sergeevna Selezneva**, Rostov Regional Children’s Clinical Hospital

**Donetsk Region**

- **Elena Alexandrovna Ovchinnikova**, Republican Specialized Center for Medical Genetics and Prenatal Diagnostics

**Republic of Crimea**

- **Marina Valentinovna Gorda**, Republican State Healthcare Institution of the Republic of Crimea “N. A. Semashko Republican Clinical Hospital”
- **Mashkovskaya Dina Valeryevna**, Republican Children’s Clinical Hospital, Simferopol

**Sevastopol**

- **Galina Gavrilovna Kodzhaeva**, City Hospital No. 5 – Maternal and Child Health Center
- **Averina Elena Vladimirovna**, Sevastopol; Medical Center

**Republic of Dagestan**

- **Aminat Ibragimovna Gamzatova**, Republican Medical Genetic Center of the Republic of Dagestan
- **Zuleikha Alaudinovna Aliskandieva**, Dagestan Republican Children’s Clinical Hospital named after N.M. Kurayev

**Republic of Ingushetia**

- **Khalimat Borisovna Nastayeva**, Republican Clinical Perinatal Center, Nazran
- **Akhmed Muradovich Bekbotov**, Republican Clinical Perinatal Center, Nazran
- **Temirbieva Ayshet Magometovna**, Children’s Polyclinic, Nazran

**Kabardino-Balkarian Republic**

- **Emma Lyoelievna Kubekova**, Perinatal Center, Ministry of Health of the Kabardino-Balkarian Republic
- **Madina Sufyanovna Shogenova**, "Center of Allergology" of the Ministry of Health of the Kabardino-Balkarian Republic

**Karachay-Cherkess Republic**

- **Zarema Medzhidovna Abaykhanova**, Republican State Healthcare Facility “Republican Perinatal Center”
- **Kulizar Zaurbekovna Bulgarova**, Republican Children’s Clinical Hospital

**Republic of North Ossetia–Alania**

- **Inna Soslanbekovna Tebiyeva**, Republican Children’s Clinical Hospital, Ministry of Health of the Republic of North Ossetia–Alania
- **Yulia Valeryevna Gabisova**, Republican Children’s Clinical Hospital, Ministry of Health of the Republic of North Ossetia–Alania
- **Roza Giviyevna Dzeranova**, North Ossetian Republican Clinical Pediatric Hospital

**Stavropol Krai**

- **Ekaterina Vladimirovna Volchkova**, Stavropol Regional Clinical Consultative and Diagnostic Center
- **Lyudmila Yuryevna Barycheva**, Stavropol State Medical University, Regional Children’s Clinical Hospital

**Chechen Republic**

- **Jamilya Khamidovna Saidaeva**, Republican Perinatal Center
- **Jaina Aslanbekovna Mudaeva**, Republican Perinatal Center
- **Asya Khamidovna Ibisheva**, Republican Children’s Clinical Hospital, Grozny

**Republic of Bashkortostan**

- **Oleg Igorevich Mashkov, Republican Medical Genetic Centre**, Republic of Bashkortostan
- **Liya Razifovna Nurgalieva**, **Republican Medical Genetic Centre**, Republic of Bashkortostan
- **Elena Vladimirovna Saifullina, Republican Medical Genetic Centre**, Republic of Bashkortostan
- **Linara Rinatovna Kalmetieva**, Republican Children’s Clinical Hospital
- **Dilyara Damirovna Prolygina**, Republican Children’s Clinical Hospital

**Kirov Region**

- **Anastasia Anatolyevna Beresneva**, Kirov Regional Clinical Perinatal Center
- **Marina Mikhailovna Yershkova**, Kirov Regional Pediatric Clinical Hospital

**Republic of Mari El**

- **Tatyana Lvovna Alekseeva**, Perinatal Center of the Republic of Mari El
- **Vera Platonovna Cherepanova**, Mari El Pediatric Clinical Hospital

**Republic of Mordovia**

- **Svetlana Sergeevna Khramikhina**, Mordovian Republican Central Clinical Hospital

**Nizhny Novgorod Region**

- **Khasyanya Fatikhovna Aksyanova**, Nizhny Novgorod Regional Children’s Clinical Hospital
- **Elena Vladimirovna Timofeeva**, Nizhny Novgorod Regional Children’s Clinical Hospital

**Orenburg Region**

- **Elena Yuryevna Belyashova**, Orenburg Regional Clinical Hospital No. 2
- **Elena Alekseevna Zlodeeva**, Orenburg State Medical University
- **Anna Vladimirovna Volzhanina**, Orenburg Regional Pediatric Clinical Hospital

**Penza Region**

- **Elena Vladimirovna Shelkova**, N.N. Burdenko Penza Regional Clinical Hospital

**Perm Krai**

- **Vera Iosifovna Kurilova**, Perm Regional Children’s Clinical Hospital
- **Ekaterina Valeryevna Troitskaya**, Perm Regional Pediatric Hospital
- **Elena Sergeevna Zubova**, Perm Regional Pediatric Hospital

**Samara Region**

- **Tatyana Vladimirovna Melnikova**, V.D. Seredavin Samara Regional Clinical Hospital
- **Farid Ilshatovich Zakirov**, Samara Regional Clinical Hospital named after V.D. Seredavin

**Saratov Region**

- **Liliya Petrovna Andreeva**, Saratov Regional Children’s Clinical Hospital
- **Lyubov Valeryevna Skuchaeva**, Saratov State Medical University named after V.I. Razumovsky

**Republic of Tatarstan**

- **Zulfiya Ilsurovna Vafina**, Republican Clinical Hospital, Kazan
- **Farida Ildusovna Sibgatullina**, Tatarstan Republican Children’s Clinical Hospital
- **Almaziya Raisovna Shakirova**, Tatarstan Republican Children’s Clinical Hospital
- **Ekaterina Yuryevna Selina**, Tatarstan Republican Children’s Clinical Hospital

**Udmurt Republic**

- **Elena Valeryevna Osipova**, First Republican Clinical Hospital, Ministry of Health of the Udmurt Republic
- **Ekaterina Vladimirovna Kitova**, Republican Clinical Infectious Diseases Hospital
- **Margarita Kuzminichna Yermakova**, Izhevsk State Medical Academy

**Ulyanovsk Region**

- **Yulia Konstantinovna Kutkova**, Y.F. Goryachev Ulyanovsk Regional Children’s Clinical Hospital
- **Aleksandr Petrovich Cherdantsev**, Ulyanovsk State University, Department of Pediatrics

**Chuvash Republic**

- **Anna Viktorovna Abrukova**, Presidential Perinatal Center, Ministry of Health of the Chuvash Republic
- **Tamara Ivanovna Petrova**, Chuvash Republican Children’s Clinical Hospital
- **Nataliya Petrovna Andreeva**, Chuvash Republican Children’s Clinical Hospital

**Kurgan Region**

- **Oksana Pavlovna Gorobets**, Kurgan Regional Perinatal Center

**Sverdlovsk Region**

- **Tatiana Ivanovna Belyaeva, Clinical Diagnostic Centre «Mother and Child Healthcare»**, Yekaterinburg
- **Maria Gennadievna Sumina**, Regional Children’s Clinical Hospital, Yekaterinburg
- **Olga Sergeevna Romanova**, Regional Children’s Clinical Hospital, Yekaterinburg
- **Elena Viktorovna Vlasova**, Regional Children’s Clinical Hospital №1, Yekaterinburg

**Tyumen Region**

- **Anna Andreevna Nikiforenko**, Tyumen Regional Perinatal Center
- **Anastasia Gennadyevna Mandzhieva**, Tyumen Regional Clinical Hospital №1

**Khanty-Mansi Autonomous Okrug – Ugra**

- **Lev Nikolaevich Kolbasin**, Surgut District Clinical Center for Maternal and Child Health

**Chelyabinsk Region**

- **Galina Viktorovna Buyanova**, Chelyabinsk Regional Children’s Clinical Hospital
- **Alena Igorevna Pobedinskaya**, Chelyabinsk Regional Children’s Clinical Hospital
- **Tatyana Vasilyevna Shilova**, South-Ural State Medical University

**Yamalo-Nenets Autonomous Okrug**

- **Lyudmila Nikolaevna Bikbulatova**, Salekhard District Clinical Hospital
- **Sergey Viktorovich Kataev**, Salekhard District Clinical Hospital

**Republic of Altai**

- **Marina Nikolaevna Shestakova**, Republican Hospital, Gorno-Altaysk, Republic of Altai

**Altai Krai**

- **Alexander Mikhailovich Nikonov**, Diagnostic Center of Altai Krai
- **Natalya Viktorovna Shakhova**, Altai State Medical University

**Irkutsk Region**

- **Irina Vasilyevna Potapova**, Irkutsk Regional Clinical Hospital (Order of the Badge of Honor)
- **Tatyana Borisovna Pavlova**, Irkutsk Regional Children’s Clinical Hospital
- **Vera Mikhailovna Shinkareva**, Irkutsk Regional Children’s Clinical Hospital

**Kemerovo Region — Kuzbass**

- **Rimma Vitalyevna Olennikova**, S.V. Belyaev Kemerovo Regional Clinical Hospital
- **Svetlana Lvovna Nersesyan**, S.V. Belyaev Kemerovo Regional Clinical Hospital

**Krasnoyarsk Krai**

- **Emiliya Eduardovna Kokh**, Krasnoyarsk Regional Medical Genetic Center
- **Darya Sergeevna Zhavoronok**, Krasnoyarsk Regional Clinical Center for Maternal and Child Health

**Novosibirsk Region**

- **Yuliya Vladimirovna Maksimova**, Medical Consultation Center, Novosibirsk State Medical University
- **Elena Viktorovna Maslova**, Medical Consultation Center, Novosibirsk State Medical University
- **Svetlana Nikolaevna Isakova**, Scientific Research Institute of Fundamental and Clinical Immunology, Novosibirsk
- **Daria Vladimirovna Demina,** Scientific Research Institute of Fundamental and Clinical Immunology, Novosibirsk

**Omsk Region**

- **Natalya Yuryevna Gerasimenko**, Perinatal Center, Omsk Regional Clinical Hospital
- **Svetlana Yuryevna Starikova**, Omsk Regional Children’s Clinical Hospital No. 2

**Tomsk Region**

- **Lyudmila Pavlovna Nazarenko**, Research Institute of Medical Genetics, Tomsk National Research Medical Center
- **Dmitriy Sergeevich Orlov,** Research Institute of Medical Genetics, Tomsk National Research Medical Center
- **Larisa Ivanovna Minaycheva**, Research Institute of Medical Genetics, Tomsk National Research Medical Center
- **Elena Mikhailovna Kamaltynova**, Tomsk Regional Children’s Clinical Hospital

**Republic of Tuva**

- **Aisluu Orlanovna Dulush**, Perinatal Center of the Republic of Tuva

**Republic of Khakassia**

- **Olga Kirillovna Togochakova**, Republican Clinical Perinatal Center of the Republic of Khakassia

**Amur Region**

- **Tatyana Yuryevna Pozdeeva**, Amur Regional Clinical Hospital

**Republic of Buryatia**

- **Elena Robertovna Yeryomina**, Republican Perinatal Center, Ministry of Health of the Republic of Buryatia
- **Kseniya Nikolayevna Ryakhina**, Republican Perinatal Center, Ministry of Health of the Republic of Buryatia
- **Zema Vladimirovna Bambaeva**, Children’s Republican Clinical Hospital of Buryatiya

**Jewish Autonomous Region**

- **Ekaterina Viktorovna Pastukhova**, Regional Hospital of the Jewish Autonomous Region

**Zabaykalsky Krai**

- **Ekaterina Igorevna Dyukova**, Regional Children’s Clinical Hospital

**Kamchatka Krai**

- **Tatyana Terentyevna Dmitrieva**, Kamchatka Regional Children’s Hospital

**Magadan Region**

- **Marina Ivanovna Nosok**, Magadan Regional Maternity Hospital

**Primorsky Krai**

- **Ekaterina Yuryevna Milkina**, Primorsky Regional Perinatal Center

**Republic of Sakha (Yakutia)**

- **Aitalina Lukichna Sukhomyasova**, Republican Hospital No. 1 – National Center of Medicine of the Republic of Sakha (Yakutia)
- **Kyunna Konstantinovna Pavlova**, Republican Hospital No. 1 – National Center of Medicine of the Republic of Sakha (Yakutia)
- **Oksana Afanasyevna Golikova**, Republican Clinical Hospital

**Sakhalin Region**

- **Eleonora Ivanovna Kostyrko**, Sakhalin Regional Clinical Hospital, Perinatal Center
- **Zhanna Vladimirovna Prokopceva**, Sakhalin Regional Clinical Hospital, Perinatal Center

**Khabarovsk Krai**

- **Natalya Vladimirovna Sikora**, Khabarovsk Regional Perinatal Center

**Chukotka Autonomous Okrug**

- **Irina Yuryevna Anisimova**, Chukotka District Hospital
